# A Case of Pacemaker Endocarditis Caused by *Aerococcus urinae*

**DOI:** 10.1155/2018/9128560

**Published:** 2018-03-05

**Authors:** Sandra Samuelsson, Charles Kennergren, Magnus Rasmussen

**Affiliations:** ^1^Department of Infectious Diseases, Hospital of Halland, Halmstad, Sweden; ^2^Department of Cardiothoracic Surgery, Sahlgrenska University Hospital, Göteborg, Sweden; ^3^Department of Clinical Sciences, Division of Infection Medicine, Lund University, Lund, Sweden; ^4^Division for Infectious Diseases, Skåne University Hospital, Lund, Sweden

## Abstract

**Background:**

*Aerococcus urinae* has lately been acknowledged as a cause of infective endocarditis (IE) especially in older males with underlying urinary tract disorders. In this population, cardiac implanted electronical devices (CIED) are not uncommon, but despite the capacity of *A. urinae* to form biofilm *in vitro*, no cases of aerococcal CIED infections have been reported to date.

**Case Presentation:**

An 84-year-old male with pacemaker was admitted with dysuria one month after a transurethral procedure for urinary bladder cancer. *A. urinae* was isolated from urine and blood. Transesophageal echocardiography (TEE) was without signs of vegetation on valves or pacing cables. The patient was treated with a twelve-day course of *β*-lactam antibiotics. Forty days after the initial admission, the patient was readmitted due to malaise, general pain of the joints, chills, and renewed blood cultures grew *A. urinae*. TEE demonstrated a 10 × 5 mm vegetation on either the tricuspid valve or one of the pacing cables. The pacemaker system was completely removed and demonstrated macroscopic signs of infection. A temporary system was implanted, and after 14 days of penicillin G treatment, a new system permanent system was implanted. Total treatment time was 40 days. Recovery was uneventful.

**Conclusion:**

This report demonstrates that *A. urinae* can cause CIED infection. In patients with *A. urinae* bacteremia and a CIED, this risk must be considered, especially if bacteremia reoccurs.

## 1. Introduction

*Aerococcus urinae* is a Gram-positive bacterium which can cause urinary tract infections (UTIs), bacteremia, and infective endocarditis (IE) [1–3]. After the introduction of MALDI-TOF MS for species determination in medical microbiology, *A. urinae* has been increasingly identified as a relatively common cause of both UTIs and bacteremia [4, 5]. Many cases of IE caused by *A. urinae* have been reported [6], but in a larger case series, this complication to bacteremia was found to occur only in around 10% of cases [5]. IE caused by *A. urinae* mainly affects older males with underlying urinary tract conditions, and the prognosis is relatively favourable despite the advanced age and many comorbidities of the patients [3, 7]. *A. urinae* has been described to induce aggregation of human platelets *in vitro* and is able to form biofilms on plastic surfaces, two potential mechanisms of importance for the pathogenesis of IE [8, 9]. A particular form of IE is an infection where bacteria adhere to the leads of transvenously coronary implanted electronical devices (CIED), such as a pacemaker. In this type of infection, adherence of bacteria to the foreign material and a subsequent formation of biofilm are believed to be important [10]. CIED infections are treated with antibiotics and device removal, a procedure not without risks. CIED infection with *A. urinae* has to our knowledge not been reported previously.

## 2. Case Presentation

An 84-year-old male presented with a medical history of sarcoma in 1999 located on the right flank (treated with surgical removal, chemotherapy, and radiation), ischemic heart disease, atrial fibrillation, and a pacemaker (St. Jude 5816, atrial lead Medtronic 4076, and ventricular lead Medtronic 4074) since 2010 due to sick sinus syndrome and AV block II Mobitz type 2. Three months prior to the present episode, he was diagnosed with urinary bladder cancer (pTaG1), and one month prior to the present admission, he underwent a diagnostic transurethral resection of bladder tissue (TUR-B).

On admission, the patient reported malaise and dysuria since the TUR-B. He was afebrile and respiratory and circulatory stable and had macroscopic haematuria, C-reactive protein (CRP) of 204 mg/L, and leukocytes of 12 × 10^9^/L. After cultures had been taken from blood and urine, he was admitted to the Infectious Diseases Clinic. On the third day, blood (4/4 bottles) and urine cultures grew *A. urinae* (>10^5^ cfu/mL). Species determination was performed with MALDI-TOF MS (Bruker Microflex) with a score of 2.23. Antibiotic treatment with ampicillin 2 g × 3 was initiated. Transesophageal echocardiography (TEE) was performed, and no signs of vegetation on valves or pacing leads could be demonstrated. After five days, the patient's condition had improved, CRP was 124 mg/L, and he was discharged with amoxicillin 500 mg three times daily for another seven days.

**Figure 1:**
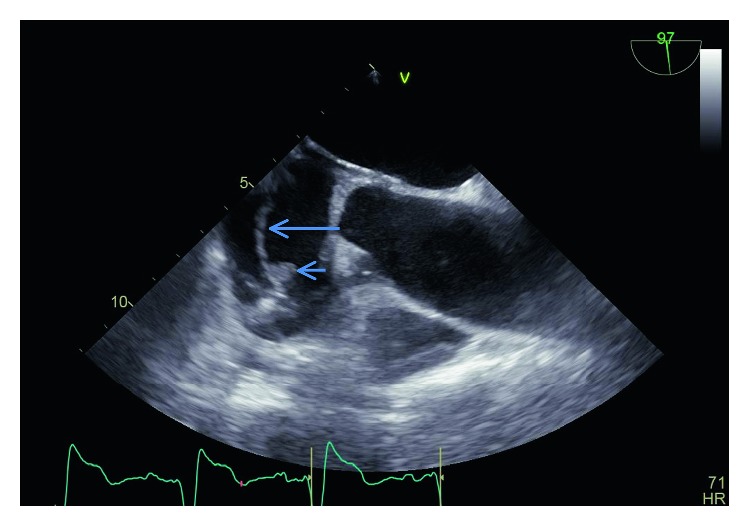
TEE demonstrating the lead (long arrow) and the vegetation (short arrow) attached to the lead.

Forty days after the initial admission, the patient was readmitted due to a two-week history of malaise and general pain of the joints. Two days before, he experienced chills but was on admission afebrile and respiratory and circulatory stable, CRP was 250 mg/L, and leukocytes were 21 × 10^9^/L. Treatment with intravenous penicillin G 3 g four times daily was initiated, and renewed blood cultures grew *A. urinae* (6/6 bottles), whereas the urine culture was negative. Minimum inhibitory concentrations were 0.008 mg/L for penicillin G, 0.25 mg/L for ceftriaxone, and 256 mg/L for tobramycin. A renewed TEE demonstrated a 10 × 5 mm vegetation on either the tricuspid valve or one of the pacing leads (Figure 1). After 19 days of treatment using penicillin G 3 g four times daily, the patient was referred to a tertiary centre for pacemaker system extraction. The extraction was performed using transvenous techniques, the device and leads were completely removed without complications, and a temporary VVI pacemaker was implanted on the ipsilateral side. The explanted electrode demonstrated macroscopic signs of infection in the form of nonfibrous material. Culture from the tip was however negative. Two weeks after extraction, a permanent dual-chamber device was implanted at the contralateral side while the patient was still treated with penicillin G.

Thirty-seven days after the second admission, the patient was discharged with ceftriaxone 2g daily for another three days. The recovery is uneventful. On follow-up 4 weeks after the end of the treatment, the patient was recovered apart from some shortness of breath during walks.

## 3. Discussion

Despite that *A. urinae* is a well-described cause of IE and that the bacterium has the potential to form biofilm on foreign materials, this is the first description of CIED infection caused by this bacterium. Upon the initial admission, this patient was subjected to TEE which did not identify vegetations on the leads. The CIED infection thus developed as a complication to the initial episode of bacteremia. Upon readmission, there was suspicion of a complicating IE or CIED infection and TEE demonstrated vegetations on one lead. The subsequent delay to extraction was due to a regional limitation in the capacity for device extraction. Lead extraction was without complications and a temporary pacemaker on the ipsilateral side was in place for two weeks before a new permanent pacemaker was implanted.

IE with *A. urinae* is typically treated with a *β*-lactam antibiotic, and we choose penicillin to which the bacterium had very low MIC [11]. Aminoglycosides are often added to the treatment despite that synergism between *β*-lactams and aminoglycosides is not always present [7, 11, 12]. In our case, MIC towards aminoglycosides was high, and cure of the patient was dependent on lead extraction rather than on antibiotic treatment. Therefore, aminoglycosides were not given.

The patient described here is prototypic for invasive infections with *A. urinae*. He is male, above 80 years old, and has urinary bladder cancer, all factors associated with *A. urinae* bacteremia [3, 5, 13]. In the first episode, the urinary tract was the likely focus of infection demonstrated by both the symptoms and bacteriuria of the patient. In the recurring episode, however, the focus of infection was rather the pacemaker system demonstrated by the vegetations on leads and the lack of bacteriuria. Cultures from the tip remained negative, but after a long course of antibiotic treatment this was the expected. In this case, the infection was macroscopically evident, but in less evident cases, 16S rRNA gene PCR and sequencing of material from leads might provide additional information.

The incidence of bacteremia with *A. urinae* has been estimated to be 1.4 per 100,000 inhabitants per year [5] and is certainly much higher among older people. The prevalence of CIED in the elderly population, at risk for *A. urinae* bacteremia, is not insignificant. Given the lack of reports about *A. urinae* CIED infection and the presumed significant number of episodes of *A. urinae* bacteremia in CIED carriers, the risk for CIED infection in *A. urinae* bacteremia is likely to be relatively low.

This case report demonstrates that *A. urinae* can cause CIED infection. In patients with *A. urinae* bacteremia and a CIED, this risk must be considered, especially if bacteremia recurs.
